# Correction: A lightweight and secure protocol for teleworking environment

**DOI:** 10.1371/journal.pone.0316745

**Published:** 2024-12-30

**Authors:** Fahad Algarni, Saeed Ullah Jan

Figs 3 to 6 are uploaded incorrectly. Please see the correct Figs 3 to 6 here.

**Fig 3:**
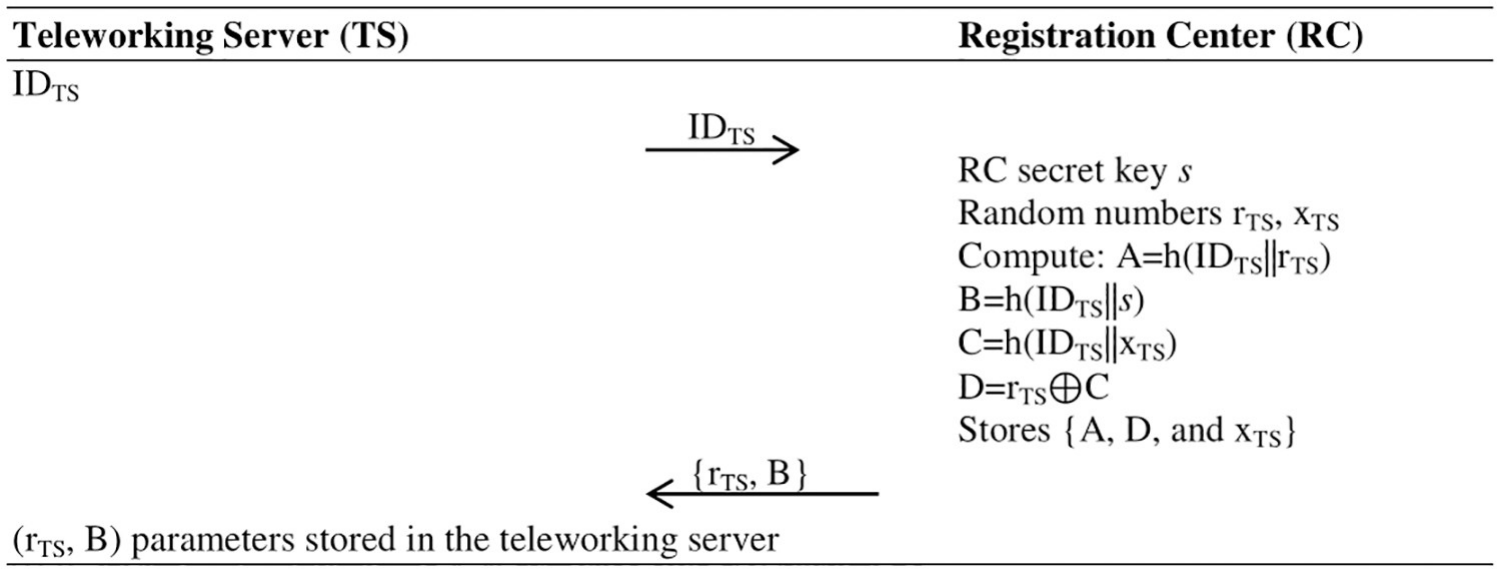
Teleworking Sever (TS) registration phase.

**Fig 4:**
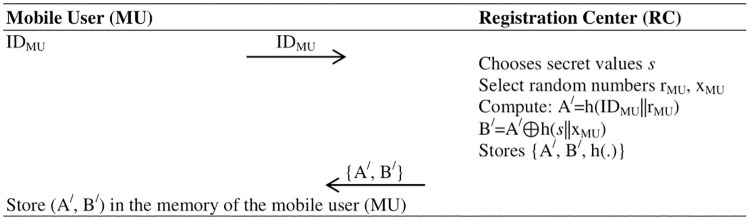
Mobile User (MU) registration phase.

**Fig 5:**
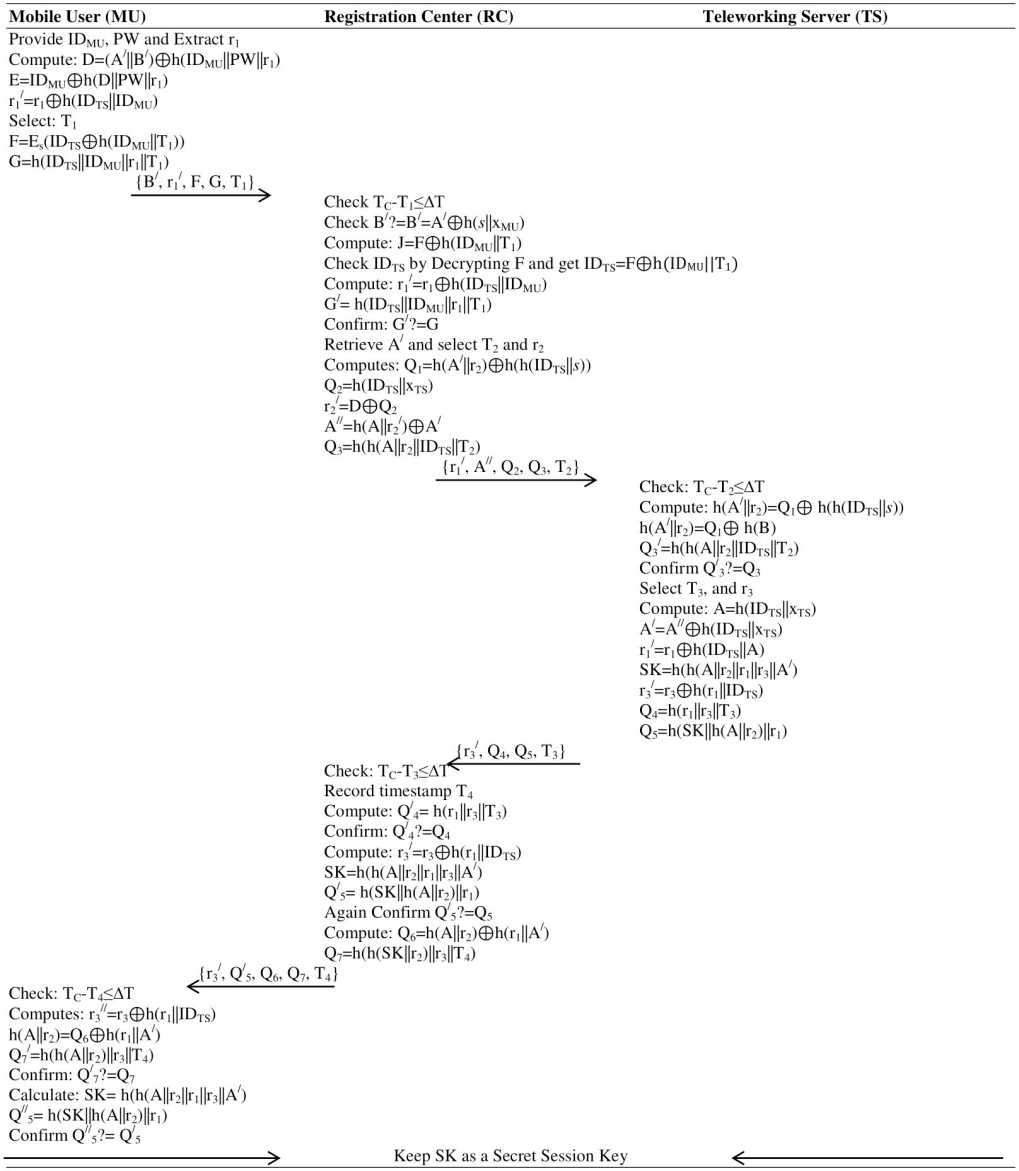
Login & authentication phase. **Remark:** The clock synchronization issue can be addressed by configuring each participant to the global clock so that it will establish the start and finish time slot as well as correct the offset and drift rate of the participants’ clock w.r.t global time.

**Fig 6:**
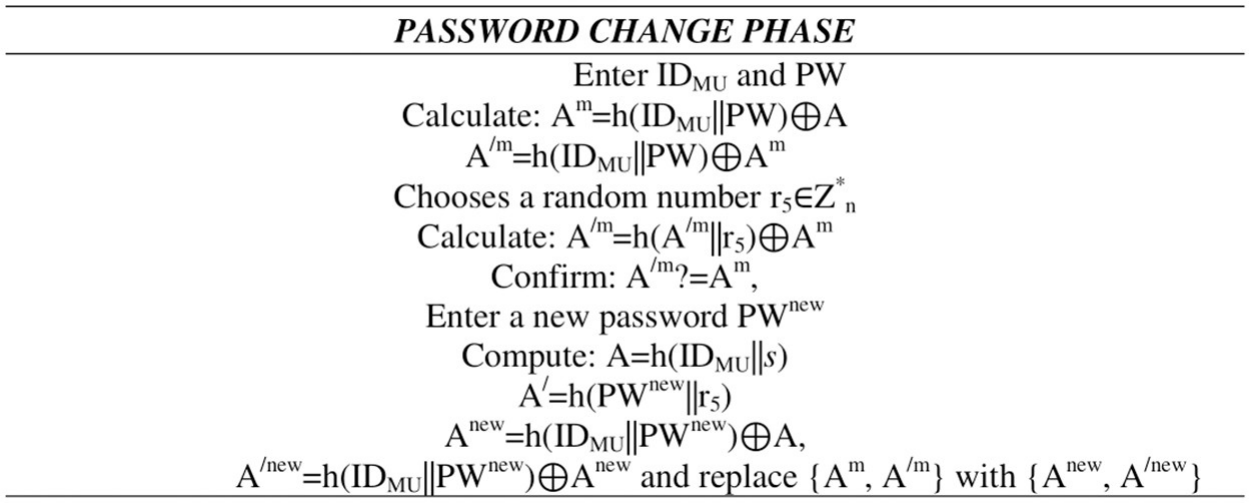
Password change phase.
