# Supplementary material for: A lightweight and secure protocol for teleworking environment
Source: PLoS One. 2024 Mar 21;19(3):e0298276. doi: 10.1371/journal.pone.0298276 (PMC10956787; doi:10.1371/journal.pone.0298276)
Supplement: Attachment — Submitted filename: comments.docx [file pone.0298276.s002.docx]

**Review Comments:**

The manuscript under the titled “**A Lightweight and Secure Protocol for Teleworking Environment**” proposed the work on lightweight and secure technique for teleworking. This study tried to resist against various attacks may be performed on the system, the comparative analysis is encouraging and sufficient.

0. Add the keywords after Abstract.

1. In Table 1, put the findings in bullets points avoid the paragraphs in table cells.

2. Construct the separate section for “Literature Review”, add more literature pertaining to 2021, 2022 and 2023. In literature you may add the following articles;

-- Meshram, C., Lee, C. C., Bahkali, I., & Imoize, A. L. (2023). An Efficient Fractional Chebyshev Chaotic Map-Based Three-Factor Session Initiation Protocol for the Human-Centered IoT Architecture. *Mathematics*, *11*(9), 2085.

-- Meshram, C., Ibrahim, R. W., Yupapin, P., Bahkali, I., Imoize, A. L., & Meshram, S. G. (2023). An efficient certificateless group signcryption scheme using Quantum Chebyshev Chaotic Maps in HC-IoT environments. *The Journal of Supercomputing*, 1-26.

-- Tembhurne, J. V., Diwan, T., & Jain, T. K. (2023). IoT Security and Privacy. In *Modern Approaches in IoT and Machine Learning for Cyber Security: Latest Trends in AI* (pp. 45-61). Cham: Springer International Publishing.

-- Meshram, C., Obaidat, M. S., Tembhurne, J. V., Shende, S. W., Kalare, K. W., & Meshram, S. G. (2020). A lightweight provably secure digital short-signature technique using extended chaotic maps for human-centered IoT systems. *IEEE Systems Journal*, *15*(4), 5507-5515.

-- Kamble, A., Gaikwad, V., & Tembhurne, J. (2023). A provably lightweight mutually authentication and key establishment protocol using extended chaotic map for telecare medicine information system. *International Journal of Information Technology*, 1-17.

3. The motivation is limited, please elaborate on this.

4. What are the research challenges you are addressing in this work?

5. What are your research questions which you are trying to solve from the proposed work?

6. Too many main points are focused in the proposed work to accomplish, justify ?

7. All equation, formulas and symbols must be math-type

8. 3.1 and 3.2 should be mentioned in points or steps then it will be more understandable.

9. In Module I and Phase –III the symbols are overlapping with arrows

10. What happens when the attacks is performed at the time of registration?

11. How the authentication is not compromised while attack? If password is revealed to the attacker, how system behaves and resist against the attack.

12. Perform the comparison with the old literature, add the latest literature i.e. 2021, 2022, and 2023 in the comparison.

13. The conclusion is not attractive and quantitative in terms of results obtained.

14. Check all the grammar errors and typos in the article.

**Accepted after major revision.**
