# Supplementary material for: A lightweight and secure protocol for teleworking environment
Source: PLoS One. 2024 Mar 21;19(3):e0298276. doi: 10.1371/journal.pone.0298276 (PMC10956787; doi:10.1371/journal.pone.0298276)
Supplement: Attachment — Submitted filename: Response to Reviewers.docx [file pone.0298276.s003.docx]

**REVIEWER #1**

**Comment 1: Please mention numerical values in the Abstract that confirm the efficiency of the proposed system.**

**Author Response: Thanks for such a valuable comment**

**Author Action: We have mentioned the maximum improvement of our protocol with its competitors in the Abstract of the article, which is discussed in detail in section 6.**

**Comment 2: No keywords were mentioned**

**Author Response: Thanks for pointing out a critical mistake**

**Author Action: In the revised, modified version of the manuscript, we have mentioned keywords after the Abstract.**

**Comment 3: Organizing the relevant research listed in the introduction according to the year of publication from oldest to newest, as is the case for references in Table 1**

**Author Response: Thanks a lot**

**Author Action: They were organized, as directed, from oldest to newest. Now, the given concern has been removed from the stated section of the article.**

**Comment 4: Comparison was made with references published within the period 2017-2021. Why was the comparison not made with a reference published during the year 2023?**

**Author Response: Great**

**Author Action: The said concern has been addressed in the newer version of the article.**

**Comment 5: Insert numerical values from the tables and figures obtained into the results to build conclusions based on more realistic values.**

**Author Response: Thanks for your valuable comment**

**Author Action: Yes, the conclusion has been revised for the improvement achieved in the proposed protocol.**

**Comment 6: Unifying the format of references, in addition to including recent references published during the year 2023.**

**Author Response: Thanks**

**Author Action: The references are revised as per journal format.**

**REVIEWER #2**

**Comment 1: Add the keywords after Abstract.**

**Author Response: Thanks for identifying it**

**Author Action:	Keywords added in the specified place of the article.**

**Comment 2: In Table 1, put the findings in bullet points to avoid the paragraphs in table cells.**

**Author Response: OK**

**Author Action:	Revised as suggested**

**Comment 3: Construct a separate section for "Literature Review" and add more literature pertaining to 2021, 2022 and 2023.**

**Author Response: Thanks**

**Author Action:	The literature Review section is mentioned as a separate one.**

**Comment 4: The motivation is limited; please elaborate on this.**

**Author Response: Thanks a lot**

# Author Action: Motivation is included before the contribution and at the end of the introduction section. Yellow text highlighted paragraph represents motivation.

**Comment 5: What are the research challenges you are addressing in this work?**

**Author Response: Thanks**

**Author Action: Research challenges are also mentioned after motivation with contributions.**

**Comment 6: What are the research questions which you are trying to solve from the proposed work?**

**Author Response: Thanks**

**Author Action:	We have given questions after the contribution statements.**

**Comment 7: Too many main points are focused on in the proposed work to accomplish. Justify?**

**Author Response: Thanks**

**Author Action: The main focus of this research work is to authenticate all the participants/critical resources of the teleworking environment securely, which means securing the teleworking environment.**

**Comment 8: All equations, formulas, and symbols must be math-type**

**Author Response: Thanks**

**Author Action: All the equations have been revised as suggested.**

**Comment 9: 3.1 and 3.2 should be mentioned in points or steps. Then, it will be more understandable.**

**Author Response: Thanks**

**Author Action: The said section (which means section 3) has been merged into section 2 (Literature Review) and mentioned in points/steps as suggested.**

**Comment 10: In Module I and Phase –III, the symbols overlap with arrows**

**Author Response: Thanks**

**Author Action: The specified overlapping on arrows has been addressed, and now the newer modified version is free of such anomalies.**

**Comment 11: What happens when the attacks are performed at the time of registration?**

**Author Response: Great**

**Author Action: Registration is performed in offline mode and over a private channel. Attacks are not possible at the time of registration. It is worth mentioning that we will typically deal with a privileged user/process, so the process is tampered with safely.**

**Comment 12: How is the authentication not compromised during the attack? If a password is revealed to the attacker, how does the system behave and resist the attack?**

**Author Response: Thanks**

**Author Action: We have described the suggested concern in section 6, subsection 3, clause 8.**

**Comment 13: Perform the comparison with the old literature; add the latest literature, i.e. 2021, 2022, and 2023 in the comparison.**

**Author Response: Thanks**

**Author Action: We have compared with different state-of-the-art works, including 2021, 2022 and 2023 publications. This means that the concern has been addressed up to a maximum extent.**

**Comment 14: The conclusion could be more attractive and quantitative in terms of the results obtained.**

**Author Response: Thanks**

**Author Action: Revised as directed. Make the conclusion attractive to readers. What we have concluded is clearly mentioned in the "Conclusion" section of the article.**

**Comment 15: Check all the grammar errors and typos in the article**

**Author Response: Thanks**

**Author Action: The revised, modified version is free of grammatical and typos mistakes.**

**REVIEWER #3:**

**Comment 1: The section number for the introduction needs to be specified. Clarify this for better reference.**

**Author Response: Thanks**

**Author Action: The modified version is free of such silly mistakes**

**Comment 2: Consider revising the first paragraph of the introduction to align more closely with the research objectives.**

**Author Response: OK, Thanks**

**Author Action: Revised as suggested**

**Comment 3: The sentence, "In an age where technology is getting smarter, the schedule of the human being is equally becoming dense," is unclear. Consider revising for better clarity.**

**Author Response: Great, and thank you very much for pointing out such a mistake**

**Author Action: The sentence was revised, and making it meaningful**

**Comment 4: The author has mentioned network-enabled devices (IoT) in the introduction section. Confirm if this is the correct abbreviation and if it's appropriately introduced.**

**Author Response: OK, Thanks**

**Author Action: Corrections have been made.**

**Comment 5: The protocol is complex and lengthy. Consider simplifying without sacrificing security.**

**Author Response: Thanks**

**Author Action: The protocol consisted of the Registration phase (MU-Registration, TS-Registration), the Authentication phase and the password change phase. We have followed a standard; if we remove the password change phase, for example, then the user will face difficulty in changing their password and vice versa.**

**Comment 6: Ensure cryptographic functions are secure and comply with current standards.**

**Author Response: Thanks**

**Author Action: Once again, check each section/step, duly verify the necessary corrections made, ensure the hash function, and find it secure.**

**Comment 7: Verify the secure use of XOR operations in the protocol.**

**Author Response: Thanks**

**Author Action: Again, duly verify and make corrections. Thanks for reminding me; this is crucial for any protocol.**

**Comment 8: Thoroughly review the handling of biometrics (Gen() and Rep() functions) to prevent false rejections or acceptances.**

**Author Response: Thanks**

**Author Action: We have presented our scheme without biometrics; if we use, for example, "Lightweight," the word should never be used in the main title. Similarly, Hackers can readily tamper with stolen biometric data to produce false positives. False positives are silicone finger impressions used to get access to confidential company information, weakening biometric security.**

**Moreover, if we generate a key from biometrics using the Gen(.)/Rep(.) function, then the communication and computation costs will rise, while SHA-1 creates a 64-bit key instead of a 1024-bit key. Therefore, the Gen(.)/Rep(.) or Biometric Fuzzy Extractor method is not used in the proposed protocol.**

**Comment 9: Ensure proper time validation considering clock synchronization issues.**

**Author Response: Thanks**

**Author Action: The said concern has been addressed. Clock synchronization related to TDMC, we have given touch beneath module III. Clock Synchronization is a communication issue, while our focus is on security. TDMA is a protocol that functions in communication, not security.**

**Comment 10: Verify the security of random number generation.**

**Author Response: Thanks**

**Author Action: Mistakes in random numbers have been removed and ensure the security and consistency of random numbers.**

**Comment 11: Address vulnerabilities identified in the cryptanalysis.**

**Author Response: Thanks for identifying a critical mistake**

**Author Action: Revised as suggested**

**Comment 12: Ensure clear and consistent notation throughout the section.**

**Author Response: Thanks**

**Author Action: Ensured consistency in notations**

**Comment 13: Consider breaking down the protocol into smaller, modular components for readability.**

**Author Response: Thanks**

**Author Action: I. Registration Phase is divided into two subsections: MU Registration and TS Registration.**

**II. Authentication Phase	III. Password change Phase**

**Comment 14: Conduct a thorough security analysis, possibly using formal methods or involving a third party.**

**Author Response: Thanks**

**Author Action: Section 5.2**

**Comment 15: Ensure compliance with established security standards and best practices.**

**Author Response: Thanks**

**Author Action: We have used worldwide techniques ROM – Analysis, GNY – Analysis and ProVerif2.03 simulation.**

**Comment 16: Clearly state any assumptions about the system, environment, or adversary model.**

**Author Response: Thanks**

**Author Action: Sections 5.1 and 5.2 demonstrate the reviewer's concern.**

**Comment 16: Address practical issues like key management, secure channel establishment, and real-world constraints.**

**Author Response: Thanks**

**Author Action: Supplementary materials consisted of code containing two channels (private and public), queries, constraints, variables, functions, and events that fully address the reviewer's concern.**

**Comment 17: Ensure consistent notation throughout the section. For example, you have both RC and RC. Make sure to use a consistent notation for entities and variables.**

**Author Response: Thanks**

**Author Action: We have addressed this concern up to a maximum extent.**

**Comment 18: Simplify lengthy sentences for better clarity.**

**Author Response: Thanks**

**Author Action: Revised as directed.**

**Comment 19: Provide a more precise description for "attacker ­(SK[])" in line 462.**

**Author Response: Thanks**

**Author Action: Actually the result generated while implementing the scheme through ProVerif, we obtained the summary mentioned in section 5.3.**

**Comment 20: Provide context for how formulas and statements in Table 3 are applied in subsequent sections.**

**Author Response: Thanks**

**Author Action: To bring ease in ready/understanding of the reader or to attract the reader towards understanding the logic and how one can apply the said technique of their protocol verification/scrutinizing.**

**Comment 21: Explicitly state any specific conditions or constraints for assumptions.**

**Author Response: Thanks**

**Author Action: According to the model, the adversary has the power to enter the server internally, monitor the open communication line, and delete, insert, or modify the message, so we assume all possible attacks. For example, suppose an adversary captures a message in the login authentication scheme and tries to launch a DoS attack; due to the time thresholds and many other checks, any illegal attempt could be discarded and so on. So, keeping in view, we have informally analyzed the proposed protocol for prominent attacks.**

**Comment 22: Ensure consistent formatting for equations and queries.**

**Author Response: Thanks**

**Author Action: This issue was fully addressed in the revised, modified version of the article.**

**Comment 23: Provide a bit more context for each equation or statement for better understanding.**

**Author Response: Thanks**

**Author Action: Mainly, we have defined the notation in Table 2, then declared assumptions and goals, and gone step-by-step for the verification/achievement of goals.**

**Comment 24: Ensure consistency in Total Costs with individual costs in line number 605.**

**Author Response: Thanks**

**Author Action: We have revised the whole section**

**Comment 25: Check the consistency of the values in bits and the total costs. The values seem to be additive, but it's important to ensure correctness.**

**Author Response: Thanks**

**Author Action: We have revised the whole section, compared it with recent articles, and checked each point again and again; we hope the revised version will be free of anomalies/mistakes.**

**Comment 26: Briefly explain the significance of each security goal (G1 to G10) for better understanding.**

**Author Response: Thanks**

**Author Action: All the goals have already been described in section 3 of the article. However, we also mentioned them one by one in the specified table for reader ease.**

**Comment 27: Double-check units for Communication Costs and clarify Computation Costs.**

**Author Response: Thanks for identifying such a mistake**

**Author Action: Correction has been made.**

**Comment 28: Ensure figures are precise and correctly labelled, with separate files as indicated in the text.**

**Author Response: Thanks**

**Author Action: Done**

**Comment 29: Present visual comparative graphs that illustrate the efficiency of the proposed system in relation to existing schemes.**

**Author Response: Thanks**

**Author Action: diagrammatically represented in Fig 3, 4, 5 and 6**

**Comment 30: Consider adding a brief recap of the main findings or contributions.**

**Author Response: Thanks**

**Author Action: Explained in each subsection means the revised version contains explanations.**

**Comment 31: The mention of future work involving blockchain technology and a network security simulator is a positive addition. It provides a direction for future research.**

**Author Response: Great, thanks for a positive note**

**Author Action: Checked and found correct**

**Comment 32: Proofreading: Conduct thorough proofreading for grammatical errors and typos.**

**Author Response: Thanks**

**Author Action: The new version is free of mistakes. All necessary grammatical mistakes/sentence corrections have been made. Hope should fulfil the standard of the journal.**
