# Supplementary material for: A lightweight and secure protocol for teleworking environment
Source: PLoS One. 2024 Mar 21;19(3):e0298276. doi: 10.1371/journal.pone.0298276 (PMC10956787; doi:10.1371/journal.pone.0298276)
Supplement: Attachment — Submitted filename: Response to Reviewers (Rebuttal Letter).docx [file pone.0298276.s004.docx]

**REVIEWER #3:**

The corrections have been updated in the manuscript; however, some major corrections are still required in the article, as outlined below.

**Comment No. 01:** The article introduces numerous notations (e.g., LA1, G1, B^/^, r_1_^/^) without representation in the notations table (Table 1). It is crucial to include all notations in the table for clarity and reference throughout the article.

**Author’s Response:** Great point ---- Thanks

We have defined LA1 to LA5 and G1 to G10 in the table, while A is a computation steps for Teleworking Server and A^/^ is a computation step for Mobile User during registration phase. These notations don’t need to be defining in the table, as it explains itself in the Phase I and II respectively. Hope, sir you will accept our reply.

**Comment No. 02:** Performance Metrics Table: In Table 7 (Performance Metrics Comparison), the cost should be mentioned along with its units (e.g., bits). This addition will enhance the comprehensibility of the presented metrics.

**Author’s Response:** Appreciated Sir, Thanks for pointing out this point. We have made the requisite changes in the specified table.

**Comment No. 03:** Figures 1 and 2: Figures 1 and 2 appear blurred, necessitating a revision to improve visual clarity. Enhanced image quality will contribute to better understanding.

**Author’s Response:** Thanks

The revised figures are now in a high resolution. Addressed the important concern up to a maximum extent, and hope sir you will accept.

**Comment No. 04:** Figures 3 and 4: In Figures 3 and 4, consider either writing the x-axis in full form or placing the full form inside the graph to eliminate potential confusion.

**Author’s Response:** Ok, Revised, thanks for such a valuable suggestion.

**Comment No. 05:** Figure 2 - System Model: Provide component names in Figure 2's system model. Additionally, there is a discrepancy between mentioning "mobile user" and depicting a PC in the image. Clarification or correction is needed for consistency.

**Author’s Response:** Revised, as suggested

**Comment No. 06:** Figures 5 and 6: In Figures 5 and 6, it is essential to mention the units of the represented data. Clearly stating the units will enhance the interpretation of the figures.

**Author’s Response:** Revised as suggested

**Comment No. 07:** Goals Achievement: The authors state that Goals 1, 2, and 3 are achieved. However, the article lacks information on the accomplishment of Goals 4 to 10. Providing insights into the fulfillment of all stated goals is necessary for a comprehensive understanding.

**Author’s Response:** Thanks, revised as mentioned below.

| G4: Untraceability | Done, 5.5.9 |
| --- | --- |
| G5: Physical Protection | Yes, addressed, sub-sub-section 5.5.10 |
| G6: Resilience to Insider Threat | sub-sub-section 5.5.2 Already explained |
| G7: Mutual Authentication | The whole paper is about mutual authentication, no need to be discussed alone. The scheme is mutually authenticate each other |
| G8: Perfect Forward Secrecy | Yes, addressed, sub-sub-section 5.5.12 |
| G9: Resists Man-in-the-Middle Attack | sub-sub-section 5.5.4 Already explained |
| G10: Resists Denial-of-Service (DoS) Attack | Yes, addressed, sub-sub-section 5.5.11 |

**Comment No. 08:** Reference: The title reference is more suitable to the proposed system "Dual Authentication and Key Management Techniques for Secure Data Transmission in Vehicular Ad Hoc Networks". Consider addition to the reference section.

**Author’s Response:** Ok, included Ref [40], and properly cited.
